# Changes of anxiety in Chinese military personnels over time: a cross-temporal meta-analysis

**DOI:** 10.1186/1752-4458-8-19

**Published:** 2014-05-18

**Authors:** Zhibing Yang, Fei Cao, Huijie Lu, Xia Zhu, Danmin Miao

**Affiliations:** 1 Department of Psychology, Fourth Military Medical University, Chang Le Western Street No.169, Xi’an Shaanxi Co. 710032, China

**Keywords:** Anxiety, Cross-temporal meta-analysis, Military

## Abstract

**Background:**

Previous cross-temporal meta-analyses have demonstrated that anxiety would get severer over time. The changes of anxiety in Chinese military personnels over time remain unclear.

**Aim:**

To explore the changes of anxiety in Chinese military personnels over nearly past two decades.

**Methods:**

Studies using the Chinese version of *Spielberger’s State-Trait Anxiety Inventory* (STAI) in Chinese serviceman on active duty were primarily collected. Mean, standard deviation and sample size of each study were extracted for meta-analysis. With sample size of each study as weighted least-squares weight, we regressed the mean scores on the data collection year to evaluate changes in anxiety from 1991 to 2011. Correlations between the anxiety scores and some social indicators were also examined.

**Results:**

The final sample consisted of 45 separate studies with a total of 18,106 participants for state anxiety and 21,047 participants for trait anxiety. Both state anxiety and trait anxiety significantly increased over the past two decades. The effect sizes for state anxiety and trait anxiety were 0.88 and 0.63, respectively. Anxiety scores were significantly correlated with some social indicators (e.g., crime rate, unemployment rate) of the corresponding years or 5 years prior to the anxiety data collection.

**Conclusions:**

Some measures must be taken to tackle the problem of the rising anxiety scores. Given that Chinese military personnels are continuing to experience high levels of anxiety, it is crucial to consider the implications for mental health care and treatment. More cross-temporal meta-analyses are needed to examine the changes of mental health in Chinese military personnels over time.

## Introduction

In the past two decades, China’ economy has experienced remarkable changes. China’s total annual GDP is increasing with an average of 10% growth rate. Some social indicators such as crime rate and unemployment rate are also on the rise according to the yearbook data. These rapid changes pose particular challenges for sociologists and psychologists. Fortunately, the social medicine and psychological problems that accompany rapid social changes of contemporary China have received attention [1-3]. China’s rapid urbanisation may have important consequences for public health [2]. A series of cross-temporal meta-analyses conducted by researchers had showed that anxiety [4], depression [5], psychopathology [6] and mental health [3,7] changed a lot over time and associated with the changes of environmental factors including overall threat (e.g., crime rate), economic conditions (e.g., unemployment rate), and social connections (e.g., divorce rate). Besides, researchers suggested that social changes might be expected to lead to rising suicide rates [8]. Moreover, a meta-analysis conducted by Li [9] revealed that socio-family environment is one of the factors associated with suicidal behaviors in mainland China. Numerous studies demonstrated that a measure of psychological traits would changes over time. Many cross-temporal meta-analyses have been conducted to investigate the changes over time in measure of psychological traits, including anxiety [4,10], extraversion [11], self-esteem [12], children’s depression [5], locus of control [13], young people’s sexual behavior and attitudes [14], social desirability [15], ego [16], IQ [17], psychopathology [6], narcissistic personality [18], dispositional empathy [19], and mental health [3,7]. In the method of cross-temporal meta-analysis, researchers correlate the mean scores on a measure with the year of data collection, weighting for sample size, to assess changes over time on particular measures (e.g., Twenge [4]). Previous studies have demonstrated that anxiety would get severer over time in American college students [4] and Chinese adolescents [10]. Some studies [1,4,10] suggested that environmental factors including overall threat, economic conditions, and social connections may have significantly impact on individuals’ anxiety levels. Previous research has shown that anxiety scores do change as a function of social factors [4]. As for military personnels, only one study regarding changes scores in the Symptom Checklist 90 (SCL-90) of members of the Chinese army was conducted [3]. Contrary to the decreased of anxiety in American college students and Chinese adolescents, Yi et al. [3] found that the mental health of Chinese army were getting better and better over time. Yet, such researches on military have rarely been carried on to evaluate the changes of anxiety over time.

The Chinese armed forces have undergone various changes, including the Millions of Disarmament in eighties and nineties, and the missions had new targets as well. The Chinese armed forces not only have to defend the motherland to guarantee people’s normal life, but also have the duty to participate in the rescue and stabilizing missions which make them face with all kinds of threats. The Chinese military personnels often have to fight at the front providing disaster relief such as in the 1998 Changjiang River flood, the 2008 Wenchuan Earthquake and the Yushu Earthquake in 2010 as well as Ya’an Earthquake in 2013. Unlike people living in common surroundings, the military environment is quite different and Chinese military personnels are likely to confront more threats. Additionally, some researchers have labeled the twentieth century “the age of anxiety” (e.g., [4,20]). These assertions suggest that fierce competition of modern life produces high levels of anxiety. Taken all these findings together, we may conclude that anxiety level may increase in general populations. To our knowledge, only one study about psychological traits changes in military personnels was found. However, Yi and his colleagues [3] found that the scores of SCL-90 in Chinese army had decreased which suggested that the mental health of Chinese army were getting better and better over time. Studies about psychological traits changes in military personnels over time are scarce and inconclusive. Thus, it is doubtful whether anxiety of Chinese military personnels would get severer over time.

To sum up, we need a cross-temporal meta-analysis to address the issue. The purpose of this study was to conduct a cross-temporal meta-analysis to explore the changes of anxiety in Chinese military personnels over nearly past two decades. Previous empirical works have led us to hypothesize that there would be a rise in anxiety of Chinese military personnels over the past two decades.

## Method

### Literature search

Studies were primarily collected through searching three of the most important Chinese academic literature databases, *Wanfang* (the Chinese Wanfang data), *CNKI* (China National Knowledge Infrastructure) and *Chongqing VIP* (Chinese Journal of Science and Technology of VIP). These three databases cover nearly all of the Chinese journals of psychology (including all related disciplines) after 1985 and most of the doctoral dissertations and master’s theses since 1995. We searched for articles using the key words *military*, *army*, *troops*, *soldiers*, *warriors*, *military officer*, *recruits*, *military students* or *armed police*, intersected with *anxiety*. All relevant studies up to January 2012 were included in present analysis.

### Inclusion criteria and moderators’ coding

**Figure 1:**
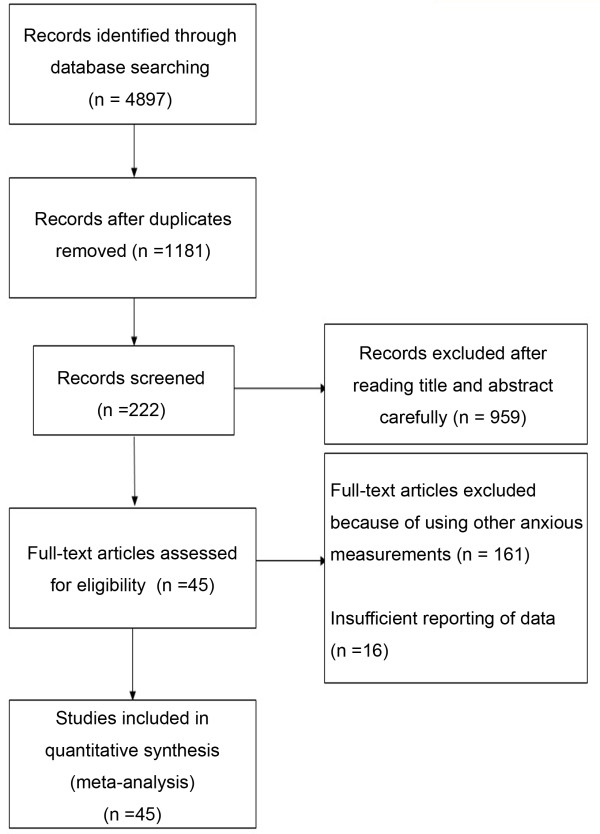
**Flowchart illustrating study inclusion and exclusion.***Note*: 31 studies containing 43 data sets for measuring both trait anxiety and state anxiety, 7 studies containing 12 data sets for measuring state anxiety, and 7 studies containing 10 data sets for measuring trait anxiety. Thus, there are 55 data sets for state anxiety and 53 data sets for trait anxiety.

The studies included in present analysis had to meet the following criteria: (a) participants were Chinese serviceman on active duty including recruits, military students or armed police; (b) the study included at least 10 male or 10 female participants; (c) participants were not clients at a counseling center or patients in hospital; (d) means were reported for unselected groups, not groups that were extremely high or low on anxious measure; (e) because the Chinese version of *Spielberger’s State-Trait Anxiety Inventory* (STAI) developed by Spielberger [21] (Chinese version is cited in Wang et al. [22]) is one of the most frequently used instruments assessing anxiety in Chinese military personnels, so we included original studies reporting anxious scores on this scale for our meta-analysis; (f) the study must report sufficient data including means, standard deviations and sample sizes. Some reports meeting these inclusion rules were excluded for the following reasons: If more than one paper using essentially the same or overlapping data were published, the report with the earliest, most complete, comprehensive results were included in the present meta-analysis. A flow diagram of the study inclusion and exclusion is shown in Figure 1. The final sample consisted of 45 separate studies with a total of 18,106 participants for state anxiety and 21,047 participants for trait anxiety. A reference list describing the 45 studies included in the meta-analysis was provided in supporting information (Additional file 1). We performed this cross-temporal meta-analysis mainly in accordance with the guidelines of the Preferred Reporting Items for Systematic Reviews and Meta-analyses (PRISMA) statement (Additional file 2) [23].

According to previous studies [4,16,24], we used the following procedure to estimate the year of data collection: (a) if the year of data collection was mentioned in the article or by the author, we included it; (b) if the article reported the original date that the article was received, we included this year as the estimated year of data collection; (c) if the article reported only the final date that the article was accepted, we subtracted this year by 1, for publication time; (d) if no other additional information reported explicitly, the year of data collection was assumed as 2 years preceding publication, as in previous studies [16,24]. For possible moderators, only publication type of studies and military categories of participants were coded. There were two types of publication studies in our meta-analysis, journal papers and doctoral dissertations or master’s theses. We code doctoral dissertations and master’s theses as one type of publication study due to a lower number of them. According to China’s military service system, military categories of participants include army, navy, air forces, second artillery force and military students. We included studies regarding military students for the following reasons. First, military students are members of Chinese military personnels in the terms of China’s military service system. Second, in China, most of military students are selected from combat troops to military college for continuing education. Other information was insufficiently reported in original studies due to military secret.

### Data analytic strategy

Mean, standard deviation and sample size of each study were extracted for meta-analysis. Where a paper reported anxious scores on the normal group before and after treatment (e.g., psychological education), only the baseline (untreated if possible) data were used.

Changes of anxious scores in Chinese military personnels over time were examined by correlating mean scores with the year of data collection. According to previous cross-temporal meta-analyses, means were weighted by the sample size of each study. We performed our analyses using Stata software, version 12.1 (Stata Corp., College Station, Texas), and the βs reported were standardized to allow for easier interpretation. To calculate the magnitude of changes in anxious scores over time, we used the regression equations and the averaged standard deviation of the individual samples when they were available. To compute the mean scores for a specific year (e.g., 2004), we used the regression equation from the statistical output, which followed the algebraic formula *y* = B*x* + C, where B = the unstandardized regression coefficient, *x* = the year of data collection, C = the constant or intercept, and *y* = the predicted mean anxious score. We computed the average standard deviation by averaging the within-sample standard deviations reported in the data sources. According to previous studies [25], this method avoids the ecological fallacy, also known as alerting correlations. To make the results to be easier understood, we converted magnitude of change into percentile scores and we used standard deviation to measure the magnitude of change of anxiety scores. We assume that anxiety scores of Chinese military personnels is a normal distribution. For example, we would refer the anxiety score in 1991 was the baseline norm (in the 50th percentile). If the anxiety score was increase one standard deviation from 1991 to 2011. According to the standard normal curve, the percentile score of anxiety score in 2011 was 84.13% (by checking the standard normal distribution table). Xin et al. [10] have utilized this method to make the magnitude of change to be informative. Finally, we converted the effect size into year-of-publication effect based on the formula r^2^ = d^2^ ÷ (d^2^ + 4) [26], which denoted the variance explained by year. We can examine the effect of sociocultural environment factors over time beyond genetics and their family environment through the variance explained by year [10,26].

### Sources for social indicators

Considering the social indicators used in Twenge’s study [4], the social indicators chosen were divorce rate, crime rate, percapita health expenditure, unemployment rate, military expenditure, promotion rate of senior school graduates. Divorce rate and crime rate come from the *China Statistical Year book*[27]; per capita health expenditure were obtained from the *China Health Statistical Year book*[28]; unemployment rate were obtained from the *China Statistical Abstract*[29]; military expenditure were obtained from the *National Defense White Paper of China*[30]; promotion rate of senior school graduates were obtained from the website of the Ministry of Education [31].

## Results

### Correlations between mean scores of anxiety and year

**Figure 2:**
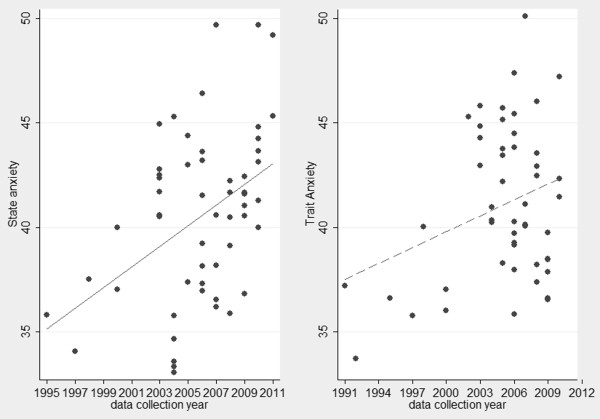
**Scatterplot and linear fit of anxiety score in Chinese military personnels, 1995–2011 for state anxiety, and 1991–2010 for trait anxiety.***Note*: The linear fit was not weighted for sample size, just for a glimpse of the gross changes pattern of anxiety score.

For a glimpse of the gross changes pattern of anxiety score, scatterplot and linear fit of anxiety score in Chinese military personnels is shown in Figure 2. The results showed that both state and trait anxiety scores increased roughly year by year. For state anxiety, when military categories of participants and publication type of studies were controlled and sample size was weighted, the correlation coefficient between state anxiety score and year of collection was 0.39, *p* = 0.0035; if these variables were not controlled and sample size was weighted, the correlation coefficient was 0.35, *p* = 0.009. As for trait anxiety, when controlling variables were controlled and sample size was weighted, the correlation coefficient between trait anxiety score and year of collection was 0.38, *p* = 0.0055; if these variables were not controlled and sample size was weighted, the correlation coefficient was 0.31, *p* = 0.0256.

The results of correlation analysis showed that Chinese military personnels’ anxiety increased with time. To know how much anxiety increased from these results, we conducted a weighted linear regression. The main analysis was a regression with year as the independent variable, mean as the dependent variable, sample size of each sample as the WLS weight, and military categories of participants and publication type of studies as potential moderators.

For state anxiety, the regression model summary is showed in Table 1. The regression equation (state anxiety mean = 0.45 × year-860.55) yielded a score of 37.20 for 1995 and 44.40 for 2011. The difference between the mean for the first and last years divided by the average SD yielded an effect size relevant for understanding differences among individuals (see also [11]). Considering the average state anxiety standard deviation of 8.20, there was a rise of 0.88 standard deviations over time. According to Cohen’s guidelines [32], it is a large effect size (medium = 0.50 and large = 0.80). To make the results to be easier understood, we converted magnitude of changes into percentile scores. If the average Chinese military personnels in 1995 scored at the 50th percentile of the distribution, the average Chinese military personnels in 2011 scored at the 81th percentile (assuming a normal distribution). On The Other Side, about twenty percent of Chinese military personnels are above the 1995 state anxiety means. Then, we converted the effect size (0.88) into variance explained by year based on the formula r^2^ = d^2^ ÷ (d^2^ + 4) [26], and found the proportion was 16%.

**Table 1:** Multiple regressions of predictors of state anxiety, weighted for sample size

**Predictors**	**State anxiety with controls**			**State anxiety without controls**		
	***B *** **(95% *CI *)**	***β***	***t***	***B *** **(95% *CI *)**	***β***	***t***
Year	0.43 (0.04 ~ 0.82)	0.33	2.20*	0.45 (0.12 ~ 0.78)	0.35	2.71**
Military categories^a^						
Navy	-2.58 (-9.87 ~ 4.70)	-0.09	-.071			
Air Forces	-0.72 (-4.61 ~ 3.16)	-0.08	-0.37			
SAF	-1.48 (-5.54 ~ 2.57)	-0.12	-0.74			
Armed Police	-1.82 (-7.64 ~ 4.00)	-0.12	-0.63			
Military Students	-2.36 (-7.04 ~ 2.32)	-0.17	-1.02			
Unknown	2.01 (-1.38 ~ 5.41)	0.27	1.20			
Publication type	-0.36 (-3.80 ~ 3.09)	-0.03	-0.21			

Note: ^a^Dummy variable was created with Army as the reference group; SAF = Second Artillery Force; *B* denotes the unstandardized regression coefficients; *β* denotes the standardized regression coefficients; *n* = 55; *p < 0.05, **p < 0.01.

As for trait anxiety, the following results showed a similar pattern that state anxiety did (Table 2). The regression equation (trait anxiety mean = 0.26 × year-482.40) yielded a score of 35.26 for 1991 and 40.20 for 2010. Considering the average state anxiety standard deviation of 7.78, the effect size was 0.63, and this is a medium to large effect size (medium = 0.50 and large = 0.80) by Cohen’s guidelines. Converting the magnitude of changes in trait anxiety to percentile scores is also informative. If the average Chinese military personnels in 1991 scored at the 50th percentile of the distribution, the average Chinese military personnels in 2010 scored at the 74th percentile (assuming a normal distribution). In other words, about a quarter of Chinese military personnels are above the 1991 trait anxiety means. As for variance explained by year, the proportion was 9%. Unlike the pattern of state anxiety, we noticed that the unstandardized regression coefficient of military students and second artillery force were higher than other military categories, which indicated that the trait anxiety score mean of military students and second artillery force were higher than army (the reference group).

**Table 2:** Multiple regressions of predictors of trait anxiety, weighted for sample size

**Predictors**	**Trait anxiety with controls**			**Trait anxiety without controls**		
	***B *** **(95% *CI *)**	***β***	***t***	***B *** **(95% *CI *)**	***β***	***t***
Year	0.24 (0.04 ~ 0.44)	0.28	2.46*	0.26 (0.03 ~ 0.49)	0.31	2.30*
Military categories^a^						
Navy	-1.85 (-4.92 ~ 1.20)	-0.15	-1.22			
Air Forces	-3.23 (-5.67 ~ -0.80)	-0.39	-2.68*			
SAF	-7.87 (-11.18 ~ -4.55)	-0.64	-4.78***			
Armed Police	-7.08 (-10.65 ~ -3.51)	-0.47	-4.00***			
Military Students	-8.04 (-11.23 ~ -4.87)	-0.65	-5.10***			
Unknown	-4.30 (-6.93 ~ -1.68)	-0.55	-3.30**			
Publication type	0.15 (-1.72 ~ 2.01)	0.02	0.16			

*Note:*^a^Dummy variable was created with Army as the reference group; SAF = Second Artillery Force; *B* denotes the unstandardized regression coefficients; *β* denotes the standardized regression coefficients; *n* = 53; *p < 0.05, **p < 0.01, ***p < 0.001.

### Correlations of Chinese military personnels’ anxiety with social indicators

The results depicted above demonstrated that anxiety level of Chinese military personnels had increased substantially over time. Yet, why the anxiety scores would increase over time? Correlating anxiety score with social indicators may provide a view of possible causes of the rise in anxiety scores of the Chinese military personnels. Several aspects of the social indicators (e.g., crime rate, unemployment rate) were matched with anxiety scores to show which aspects of the environment were linked with changes in anxiety. The social indicators were matched with the anxiety data in two ways: five years before the data were collected, and the year of data collection. These correlations are shown in the Tables 3 and 4. These tables reported correlations among the anxiety scores, social indicators collected and the Z-scored scale combination of social indicators. The results showed that some part of correlations were significant. For state anxiety, most social indicators preceding 5 years were significantly correlated with state anxiety (*p*s < 0.05). However, none of the social indicators preceding 5 years were significantly correlated with trait anxiety (*p*s > 0.05). The strongest correlations appeared between the Z-scored scale combination of social indicators and anxiety in the actual year (*ps* < 0.01). The correlations coefficients between Z-scored scale combination of social indicators and state anxiety and trait anxiety were *r* = 0.50 and *r* = 0.68, respectively (*p*s < 0.01).

**Table 3:** Correlations between social indicators and state anxiety score of Chinese military, weighted for sample size, 1995–2011

**Social indicators**	**5 years prior**	**Actual year**
*Economic conditions*		
Unemployment rate	0.24	0.28*
Military expenditure	0.27*	0.24
*Overall threat*		
Crime rate	0.31*	0.50**
Per capita health expenditure	0.29*	0.17
PRSSG	0.36**	0.26*
*Social connectedness*		
Divorce rate	0.10	0.22
*Z* total	0.26^a^	0.50**

*Note:* PRSSG = Promotion Rate of Senior School Graduates; *Z* total was a combined *Z* score of all the social indicators; *n* varies from 35 to 55 because of missing value of social indicators for some years; *p < 0.05, **p < 0.01, ^a^*p* = 0.055.

**Table 4:** Correlations between social indicators and trait anxiety score of Chinese military, weighted for sample size, 1991–2011

**Social indicators**	**5 years prior**	**Actual year**
*Economic conditions*		
Unemployment rate	-0.17	0.45***
Military expenditure	-0.15	0.06
*Overall threat*		
Crime rate	0.01	0.70***
Per capita health expenditure	-0.09	0.03
PRSSG	0.05	0.46***
*Social connectedness*		
Divorce rate	-0.23	0.11
*Z* total	-0.15	0.68***

*Note:* PRSSG = Promotion Rate of Senior School Graduates; *Z* total was a combined *Z* score of all the social indicators; *n* varies from 38 to 53 because of missing value of social indicators for some years; ***p < 0.001.

## Discussion

By examining data collected from 45 studies over the past two decades, we were able to evaluate changes of anxiety in Chinese military personnels from 1991 to 2011 and to compare these changes to social indicators. As expected, the finding of present study showed that state anxiety and trait anxiety had rise significantly over the past two decades (0.88 and 0.63 standard deviation, respectively). Besides, we found significantly correlations between anxiety scores and environmental factors including overall threat, economic conditions, and social connections. A cross-temporal meta-analysis conducted by Xin [1] found an increase of anxiety in Chinese College school students. Additionally, the finding from present study is consistent with earlier studies indicating an increase of anxiety in American college students over time [4,10]. Twenge’ study [4] suggested that environmental factors including overall threat, economic conditions, and social connections may have significantly impact on individuals’ anxiety levels. Xin [1,10] found the same pattern in Chinese middle school students and adolescents. Besides, our results indicated that the sociocultural environment might have significant effect on the anxiety of Chinese military personnels beyond genetics and their family environment [4,19]. Taken all these findings together, we may speculate that the sociocultural environment might have significant effect on the anxiety of people inside of military context and outside of it. In our study, both state anxiety and trait anxiety were increasing over the past two decades, and effect sizes for state anxiety and trait anxiety were 0.88 and 0.63, respectively. According to Tables 1 and 2, the slopes of regression equations of predicting state anxiety and trait anxiety were 0.45 and 0.26, respectively (without controls, unstandardized coefficient). These results indicate that state anxiety is increasing greater than trait anxiety. Perhaps this is because state anxiety is not so stable, and is more vulnerable to external environment. However, trait anxiety is a kind of personality trait, and not easily affected by external environment as state anxiety. State anxiety is temporary, and trait anxiety is a stable part of personality [21,33-35]. Noticing that trait anxiety score mean of military students and second artillery force were higher than army (the reference group), the sample collected is heterogeneous to some extent, and the trait anxiety would increase with controls. If the persistent trend of changes of anxiety last, the anxiety level of Chinese military personnels will continue to rise. This should cause the attention of the military and the related departments.

Yet, a vast amount of social changes have occurred simultaneously in China over the past two decades. We also examined the relationship between anxiety and environmental factors including overall threat, economic conditions, and social connections. In present study, some social indicators had a significant relationship with anxiety, and the strongest correlations appeared between the Z-scored scale combination of social indicators and anxiety in the actual year. Overall threat can be evaluated using environmental indicators such as crime rates, fear of diseases, per capita health expenditure, and fear of terrorism. Consistent with previous studies [1,4,10], our study reveals that indicators of overall threat are associated with increased anxiety, especially with state anxiety. Overall threat indicates a dangerous outside environment and may increase the anxiety by creating a sense of danger. For example, fear of victimization may be created by crime, thus anxiety is induced. Unemployment rate and military expenditure are indicators of economic conditions, and have a significant relationship with higher levels of depression and anxiety [36]. Despite the increasing of military expenditure of China, Chinese military personnels gets a relative low wage than military personnels of most developed countries (e.g., The USA). Although the allowance has been gradually raised up, economics of China is in a state of inflation, and the consumer price index (CPI) substantial rose up in recent years. Additionally, the rise of unemployment rate may gain the anxiety of Chinese military personnels when they confront their retirement and secondary employment. Nevertheless, somewhat differing from previous study [4], divorce rate was not significant associated with increased anxiety. Noticing that, the population of China has increased sharply over the past several decades. The divorce rate in China seemed increasing slowly during the past two decades with a minimum, maximum and mean as 0.88‰, 2.00‰, and 1.43‰ respectively. Another reason for the null results of association between anxiety and divorce rate may be that there are items for special protecting servicemen’s marriage in “*Marriage Law*” of China. The divorce rate of Chinese military personnels may be lower than the overall populations’. Additionally, the military people are generally young and not married and the social indicators utilized for this study were representative of the entire China. Thus, the divorce rate in China may not link to Chinese military personnels’ anxiety. Some other indicators of social connections should be needed to examine the relationship between them and anxiety. Although the Chinese armed force is gradually increased foreign exchange and more and more open and transparent, military context is a relative close environment to some extent, and some other indicators related to military context should be used in studies to examine the relationship between them and anxiety. However, other indicators were unavailable due to military secret. Although the social indicators utilized for present study were representative of the entire China, according to previous research [3,4], the social indicators in present study were conventional necessary to evaluate the changes of sociocultural environment factors. For instance, divorce rate can reflect social connectedness; unemployment rate can reflect economic conditions and crime rate can reflect overall threat. The Chinese military personnels connected more and more with civil society, and social indicators in present study may be the best data available so far. Some limitations in the current study should be addressed. First, only studies using STAI were collected in our across-temporal meta-analysis. Future researchers are advised to use other measurements of anxiety or combine them in the across-temporal meta-analysis (as Twenge [4] did). Second, someone may argue that the data measured anxiety only through self-report scales is vulnerable to social desirability. This limitation cannot be completely overcome. Besides, researchers said that self-report measures were the best data available for studying changes over time in anxiety [4]. Third, military personnels is a men dominant career. Military personnels are not necessarily representative of the overall population. Thus the generalizability of the findings may be limited. The sample collected is heterogeneous to some extent. In the terms of China’s military service system, however, army, navy, air forces, second artillery force, armed police and military students are members of Chinese military personnels. So we should include these participants in our across-temporal meta-analysis. Actually, Chinese military personnels have common military rules and regulations, administrative regulations and similar life pattern. In Chinese military context, all Chinese military personnels are under strict military and work discipline, and military training and political study are common life contents of them. Additionally, they share the common surrounding sociocultural environment. So the sample collected may broadly reflect the composition of Chinese military personnels and they have much in common. Finally, correlations between anxiety scores and social indicators cannot prove causation, but show which social indicators covary with the personality traits.

## Conclusions

Despite its limitations, the present study considerably extends our insight into the changes of anxiety in Chinese military personnels over time and which social indicators covary with anxiety scores. Moreover, the findings of present study can provide some practical implications. As anxious people predispose to depression [37], and have a higher mortality rate [38], some measures must be taken to tackle the problem of rising anxiety scores. Given that Chinese military personels are continuing to experience high levels of anxiety, it is crucial to consider the implications for mental health care and treatment.

## Competing interests

The authors declare that they have no competing interests.

## Authors’ contributions

Conceived and designed the experiments: ZY, FC, XZ, DM. Performed the experiments: ZY, FC, HL, XZ, DM. Analyzed the data: ZY, FC, XZ, DM. Contributed reagents/materials/analysis tools: ZY, FC, HL, XZ, DM. Wrote the paper: ZY, FC, HL, XZ, DM. All authors read and approved the final manuscript.

## Supplementary Material

Additional file 1 References of included studies in meta-analysis. Click here for file

Additional file 2 PRISMA 2009 checklist. Click here for file
