# Supplementary material for: Changes of anxiety in Chinese military personnels over time: a cross-temporal meta-analysis
Source: Int J Ment Health Syst. 2014 May 18;8:19. doi: 10.1186/1752-4458-8-19 (PMC4037746; doi:10.1186/1752-4458-8-19)
Supplement: Additional file 1 — References of included studies in meta-analysis. [file 1752-4458-8-19-S1.doc]

**References of included studies in meta-analysis**

1. 郭小朝, 李良明 (1993) 部队飞行员的特质焦虑分数. 中华航空医学杂志: 4.

2. 郭小朝, 李良明 (1993) 特质焦虑和飞行学员差错及技术停飞率关系的初步研究. 中华航空医学杂志: 3.

3. 黄少罗 (1997) 专业技术院校学员射击考试焦虑问题的研究. 体育学刊.

4. 谭百庆, 刘艳 (1998) 武警战士特质焦虑及相关因素. 健康心理学杂志: 3.

5. 谭百庆, 漆兵, 刘艳 (1998) 新兵首次实弹射击时应激心理研究. 中国心理卫生杂志.

6. 孙长星 徐 (2000) 新兵初始训练阶段身体素质与焦虑情绪的关系. 体育学刊: 2.

7. 艾旭, 张丽萱, 陈春杰 (2003) “非典”时期战士焦虑状况调查. 美国中华健康卫生杂志: 2.

8. 程灵芝 (2004) 急性军事应激及心理训练对新兵免疫功能的影响研究.

9. 王一牛, 郭辉, 王晓伟, 马惠, 罗跃嘉 (2004) 军人情绪状态特点与评估方法研究. 第四军医大学学报 25: 3.

10. 黄秀琴, 杨小柳, 张倩, 何晓冰, 骆利 (2005) 综合心理干预对军队献血者焦虑状态及献血反应的影响. 华北国防医药 17: 2.

11. 李敏, 汪涛, 李彦章, 张俐, 黄梅园, etc. (2005) 跳伞应激对伞兵心理情绪的影响. 中国临床康复 9: 2.

12. 王江澜 (2005) 军人心理素质训练对防空兵心理健康的影响.

13. 王煜蕙, 敖景文, 姚力萍 (2005) 高教机飞行训练诱发状态焦虑的干预治疗. 临床军医杂志 33: 3.

14. 乐燕, 刘晓虹, 江有琴, 包瀛春 (2006) 影响个体晕船发生的心理因素. 第二军医大学学报 27: 3.

15. 胡鸿群, 杨国愉, 冯正直, 张均, 王乃文, etc. (2007) 空军部队军人焦虑特点及影响因素研究. 第四军医大学学报 28: 3.

16. 刘玉华, 冯正直, 王莉, 杨国愉, 李学军 (2007) 军事飞行员应对方式、状态特质焦虑与心理健康的相关性研究. 中国行为医学科学 16: 3.

17. 王丽杰, 苗丹民, 严进 (2007) 野战医疗训练对医务人员心理状况的影响. 第四军医大学学报 28: 2.

18. 王丽杰, 严进 (2007) 维和部队参训人员心理防御方式与状态-特质焦虑的相关性分析. 中国行为医学科学 16: 2.

19. 徐蕾, 刘新, 王志慧, 王宗文 (2007) 心理训练对高特质焦虑新兵生理指标和考核成绩的影响. 中华航海医学与高气压医学杂志 14: 3.

20. 杨国愉 (2007) 青年军人特质焦虑及其认知加工特点.

21. 安婷, 程祺, 潘昱, 严进 (2008) SMMU军事应激反应性焦虑预测量表的初步编制及其预测率的研究. 中国行为医学科学 17: 3.

22. 程祺, 安婷, 严进, 袁水平, 王丽杰 (2008) 军人胃肠道症状与心理健康水平的相关性研究. 中国行为医学科学 17: 3.

23. 蒋一平 (2008) 潜艇部队新兵焦虑情绪与应对方式的关系. 中国疗养医学 17: 2.

24. 梁勇, 王丽杰, 程祺, 严进 (2008) 团体心理咨询对军事应激条件下军人应对方式和状态-特质焦虑的影响. 第二军医大学学报 29: 3.

25. 刘玉华 (2008) 军事飞行员焦虑特点及其与SCL-90和应对方式的相关研究.

26. 王雅西, 彭顺舟, 胥全宏, 宗瑛, 程波, etc. (2008) 西藏高原军人应对方式、状态特质焦虑与心理健康的相关性研究. 西南军医 10: 3.

27. 徐伦, 姜宏, 刘涛生 (2008) 军医大学学员新生军训期间焦虑状况及其影响因素研究. 第二军医大学学报 29: 3.

28. 严进 程王苗 (2008) 军人慢性心理应激对认知功能的影响. 第四军医大学学报: 3.

29. 张玉梅 (2008) 西藏武警官兵焦虑、应对方式及其关系研究.

30. 李莉, 李敏 (2009) 野战部队军人特质焦虑与社会支持的相关研究. 学理论: 2.

31. 覃时满 张吴曲苗 (2009) 基层部队官兵心理健康状况调查. 人民军医: 2.

32. 胡光涛, 贺英, 崔建新, 李学成, 陈许波, etc. (2010) 1104名新兵心理健康水平及相关因素分析. 解放军预防医学杂志 28.

33. 胡光涛, 贺英, 王军, 冯正直, 李学成, etc. (2010) 执行多样化军事任务军人心理应激的多因素研究. 西南国防医药 20.

34. 胡光涛, 王军, 贺英, 李学成, 陈静, etc. (2010) 待退役军人状态-特质焦虑、个性、应对与心理应激的相关性研究. 西南国防医药 20: 3.

35. 黄圣排 袁彭赵邓熊 (2010) 某部海训官兵心理健康水平调查与分析. 人民军医: 3.

36. 鲁娟, 李松, 李健 (2010) 新形势下军医大学心理健康教育效果研究. 中国健康心理学杂志 18: 2.

37. 王丽杰, 董兆伦, 严进, 于军, 董建树, etc (2010) 军事应激状态下不同特质焦虑者发生状态焦虑生理与生化的变化差异. 中华健康管理学杂志 04.

38. 严进 袁赵黄熊 (2010) 团体认知干预对官兵心理健康水平影响的观察. 人民军医: 2.

39. 杨海 (2010) 军校新生心理弹性因素及心理训练对心理健康影响的实证研究.

40. 叶超然, 苟仲勇 (2010) 高原汽车兵的心理健康问卷分析及服务对策. 职业与健康 26.

41. 曾昌琴, 钟代曲, 李训军, 蒋晓江, 张涛, etc. (2011) 战士48h睡眠剥夺试验方案的设计与应用. 中华现代护理杂志 17.

42. 陈良恩, 安瑞卿, 张晓丽, 张清俊 (2011) 新兵跳伞心理应激对唾液皮质醇水平的影响. 华南国防医学杂志 25.

43. 宋金华 (2011) 武警狙击手心理品质特征及心理技能训练效果研究.

44. 石文磊, 刘宇凝, 沈兴华 (2012) 汶川地震后2年救灾官兵创伤后应激障碍的发生率及其影响因素. 解放军预防医学杂志.

45. 杨军, 施旺红, 化振, 谭贤珍, 王海忠, etc. (2012) 跳伞新兵恐高反应倾向与症状、自我和谐及状态焦虑的相关研究. 中国健康心理学杂志 20.
